# Changes in soil water holding capacity and water availability following vegetation restoration on the Chinese Loess Plateau

**DOI:** 10.1038/s41598-021-88914-0

**Published:** 2021-05-07

**Authors:** Yong-wang Zhang, Kai-bo Wang, Jun Wang, Changhai Liu, Zhou-ping Shangguan

**Affiliations:** 1 Shaanxi Key Laboratory of Chinese Jujube, Yan’an University, Yan’an, 716000 Shaanxi People’s Republic of China; 2 State Key Laboratory of Soil Erosion and Dryland Farming on the Loess Plateau, Institute of Soil and Water Conservation, Chinese Academy of Sciences and Ministry of Water Resources, Yangling, 712100 Shaanxi People’s Republic of China; 3 Institute of Earth Environment, Chinese Academy of Sciences, Xi’an, 710061 Shaanxi People’s Republic of China

**Keywords:** Ecology, Hydrology

## Abstract

Changes in land use type can lead to variations in soil water characteristics. The objective of this study was to identify the responses of soil water holding capacity (SWHC) and soil water availability (SWA) to land use type (grassland, shrubland and forestland). The soil water characteristic curve describes the relationship between gravimetric water content and soil suction. We measured the soil water characteristic parameters representing SWHC and SWA, which we derived from soil water characteristic curves, in the 0–50 cm soil layer at sites representing three land use types in the Ziwuling forest region, located in the central part of the Loess Plateau, China. Our results showed that the SWHC was higher at the woodland site than the grassland and shrubland, and there was no significant difference between the latter two sites, the trend of SWA was similar to the SWHC. From grassland to woodland, the soil physical properties in the 0–50 cm soil layer partially improved, BD was significantly higher at the grassland site than at the shrubland and woodland sites, the clay and silt contents decreased significantly from grassland to shrubland to woodland and sand content showed the opposite pattern, the soil porosity was higher in the shrubland and woodland than that in the grassland, the soil physical properties across the 0–50 cm soil layer improved. Soil texture, porosity and bulk density were the key factors affecting SWHC and SWA. The results of this study provide insight into the effects of vegetation restoration on local hydrological resources and can inform soil water management and land use planning on the Chinese Loess Plateau.

## Introduction

Soil provides ecosystem services and also benefits society by producing biomass and maintaining biodiversity^1^. However, soil erosion is widespread worldwide, especially in the Loess Plateau region of China, and soil erosion has become a major environmental problem that limits the survival and development of human beings and the sustainable development of the global economy and society^2^. Soil erosion can cause soil degradation, reduce land productivity, threaten agricultural production and food security; furthermore, the environment and socioeconomic development in areas adjacent to erosion regions are affected by pollutants transported by runoff and sediment, which can cause water eutrophication and habitat destruction and intensify drought and waterlogging disasters in downstream areas^3^. Preventing and controlling soil erosion can benefit the environment, provide ecological security, and allow sustainable development and harmony between humans and nature^4^.

Vegetation restoration is an effective measure for preventing and controlling soil erosion. In recent years, with the implementation of China's national policy to return farmland to forest and grassland, soil and water loss on the Loess Plateau has decreased significantly^5^. Soil moisture, as the main factor regulating plant communities, can affect the structure and complexity of the plant community during long-term vegetation restoration and reconstruction. The change in land use following vegetation restoration can alter the root systems of plants and nitrogen fixation, which can lead to changes in the richness and composition of the soil microbial community, improve soil physical properties, and change the soil surface features. Changes in the surface properties of soil inevitably result in changes in other soil properties, such as soil water conductivity, aggregate stability, and particle composition. In addition, the transition among vegetation restoration stages can cause variations in soil water characteristics. The soil water characteristic curve describes the relationship between gravimetric water content, volumetric water content, or degree of saturation and soil suction (or equivalent relative humidity)^6^. Compared with tests in traditional soil mechanics, tests that directly measure unsaturated soil properties are not as easily accessible and are often extremely labor intensive. One tool that has made the analysis of unsaturated soil data simpler and more practical is the soil water characteristic curve^7,8^. The soil water characteristic curve can be used to indirectly determine the soil shear strength, permeability, change in water volume, water holding capacity and water availability.

The photosynthesis rate, carbon allocation, plant growth, nutrient cycling and microbial activity have close relationships with the soil water status^9^. How much water a soil can hold against gravity is very important for plant growth because the water retained in the soil can compensate for a lack of precipitation in dry years, but not all of the water held by soil is available for plant growth. However, directly assessing the available water for plant growth is challenging because of the expensive and complicated laboratory measurements required, limiting the availability of data. The soil water holding capacity (SWHC) can be used to estimate the maximum amount of water stored in the soil and reflects the capacity of the soil to provide water for plant growth. The change in land use that occurs during vegetation restoration has been identified as one of the most powerful agents driving environmental change and can explain > 50% of the variability in water quantity^10^. Vegetation type, plant species^11,12^ and the activities of fungi and bacteria^13^ also influence soil water characteristics. Deforestation and cultivation may cause a decrease in soil infiltration by decreasing porosity and SWHC. Furthermore, changes in land use type may cause changes in the physical and chemical properties of soil and in the soil microbial community^14^. Previous research conducted in the 0–20 cm soil layer in different vegetation types in the hilly gullied region of the Loess Plateau revealed that the soil water retention curves of all vegetation types exhibited approximate "S" shapes. The ranges of soil available water in the 0–10 cm and 10–20 cm soil layers were 22.65–26.80% and 23.97–28.13%, respectively, among the different vegetation types, and the soil moisture availability of grass communities and perennial *Artemisia* communities was greater than that in the annual herbaceous community and less than that of the shrub communities except the *Bothriochloa ischaemum* community and the *Robinia pseudoacacia* forest. Furthermore, in the 10–20 cm soil layer, the soil water capacities of the grass communities and perennial *Artemisia* communities were higher than that of the annual herbaceous community and lower than that of the shrub community^15^. However, the variation and potential of SWHC and the corresponding influencing factors at different vegetation restoration stages from grassland to shrubland to woodland on the Chinese Loess Plateau are unknown.

All water remaining in the root zone reservoir cannot be taken up by the plant as rapidly as needed because it is held too tightly by the soil particles^16^. Soil water availability (SWA) reflects the dynamic changes of several soil characteristics under the comprehensive actions of atmospheric, crop and soil factors; it can indicate whether soil water can be used by crops and, if so, how readily. SWA is one of the most important factors used to study the soil water environment. Plant roots, soil temperature, plant growth, microbial respiration, substrate availability, stomatal conductance, transpiration and belowground C allocation all have interaction effects with SWA^17^. Nevertheless, information about the responses of soil properties (bulk density (BD), porosity and texture) to SWA and SWHC at different vegetation restoration stages on the Chinese Loess Plateau is currently unknown. Thus, in this study, we hypothesized that the SWA and SWHC varied with long-term natural vegetation restoration ages throughout succession, and the specific aims of the present study were to examine the variation in the soil water characteristic curve at different stages of vegetation restoration, investigate the key soil properties affecting SWHC and SWA and assess the effects of different vegetation restoration stages on SWHC and SWA.

## Materials and methods

### Study area

**Figure 1:**
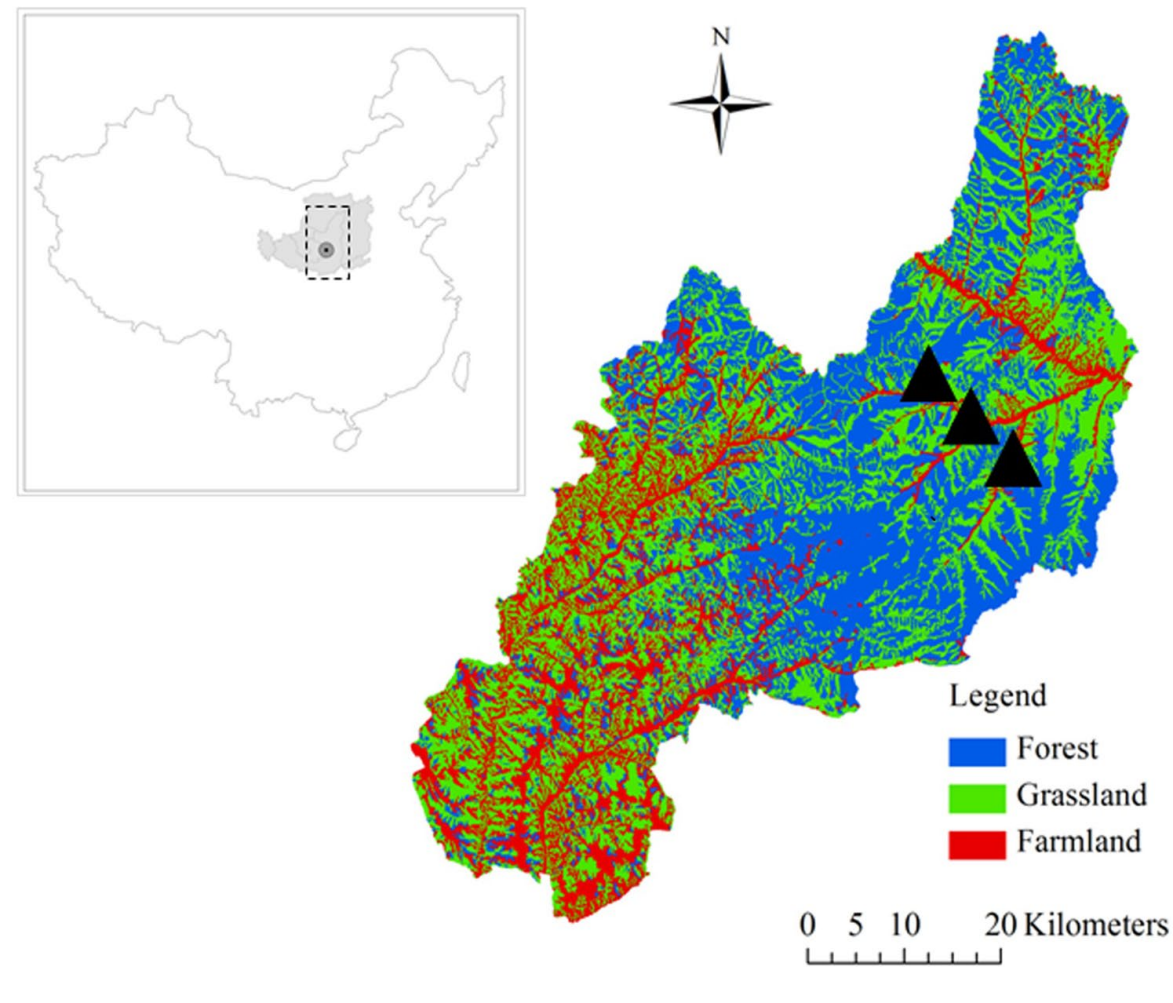
Land use map in the study area. It was created using ArcGIS version 10.6, from ArcGIS Software, Inc., Esri USA, https://support.esri.com/en/products/desktop/arcgis-desktop/arcmap/10-6.

**Figure 2:**
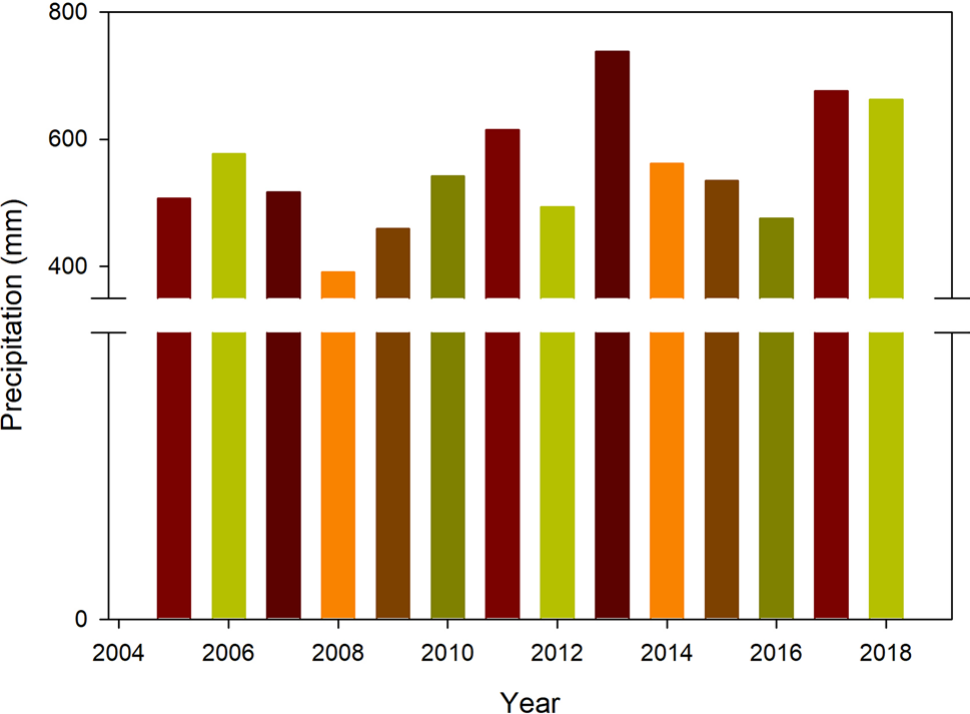
Precipitation in the study area.

The Ziwuling forest area occupies approximately 23,000 km^2^ and is the sole secondary forest region remaining on the Chinese Loess Plateau^18^. An intact series of naturally recovering vegetation succession is present on the Loess Plateau. We chose the Ziwuling Forest Region of the Chinese Loess Plateau as the study area to assess the SWHC and SWA of the soil reservoir. Information about the responses of soil properties to SWHC and SWA at different vegetation restoration stages remains lacking. Soil properties, including soil BD, soil texture and soil porosity, etc., are key factors influencing soil water characteristics. For this reason, in our study, we used land use and land cover change (LUCC) over time and adopted some simple assumptions about its impacts on soil water characteristics under a range of vegetation restoration stages. We hypothesized that vegetation restoration stage significantly affects soil BD, porosity, texture and water content and consequently influences soil water characteristics, such as SWHC and SWA. The Lianjiabian Forest Farm (35° 03′–36° 37′ N, 108° 10′–109° 18′ E) in eastern Gansu Province is in the central part of Ziwuling forest region, with an area of 23,000 km^2^ and an altitude of 1211–1453 m above sea level (asl) (Fig. 1), figure legend was created using ArcGIS version 10.6, from ArcGIS Software, Inc., Esri USA, https://support.esri.com/en/products/desktop/arcgis-desktop/arcmap/10-6.The dominant soil type is loessal mein soil (Calcaric Regosol, FAO/UNESCO, 1988). The annual mean precipitation of the area is 587 mm, and there is significant seasonal variation in precipitation (Fig. 2). The land uses in the study area include grassland, shrubland and woodland, and natural vegetation restoration has occurred from grassland to shrubland to woodland over a period of approximately 160 years^19^. The recovery times were estimated by counting growth rings and consulting related written sources^20^. The main species at the different vegetation restoration stages are listed in Table 1.

**Table 1:** Geographical and vegetation characteristics at different vegetation restoration stages in the Ziwuling forest region of the Loess Plateau.

Vegetation restoration stage	Latitude (N)	Longitude (E)	Altitude (m)	Aspect	Slope (°)	Coverage (%)	Main plant species
G (30 a)	36° 05′ 08.8″	108° 31′ 38.9″	1365	NE	8	85	*B. ischaemum*
S (50 a)	36° 04′ 14.4″	108° 32′ 01.4″	1354	NE	18	90	*H. rhamnoides*
W (160 a)	36° 02′ 57.5″	108° 32′ 13.7″	1449	NE	18	95	*Q. liaotungensis*

G represents grassland, S represents shrubland, and W represents woodland. The numbers in parentheses are the years since cropland abandonment.

### Soil sampling and analysis

Three soil sites (in grassland, shrubland and woodland) were selected, there are five duplicates for grassland, shrubland and woodland. The grassland plots were 2 × 2 m, the shrubland plots were 5 × 5 m, and the woodland plots were 20 × 20 m. Soil samples were collected at five depths below the soil surface (10, 20, 30, 40, and 50 cm) in each plot. To construct the soil water characteristic curves under various suction states (0, 0.1, 0.2, 0.4, 0.6, 0.8, 1.0, 1.5, 2.0, 4.0, 6.0, and 10.0 bar), samples were first saturated for 24 h and then weighted to determine the soil water content at saturation before submitting them to water extraction by centrifugation^21^ with a Hitachi CR21G centrifuge at a temperature of 20 °C. Soil BD was measured using the oven-dried weight method with the core sampler^22^. The soil saturated moisture content, field moisture capacity and wilting water content were used to calculate the total porosity, inactive porosity, aeration porosity and capillary porosity^23^.

### Soil water characteristic parameters

The soil water characteristic parameter A obtained from the soil water characteristic curves was calculated by the following power function empirical equation:

$$ \theta = AS^{ - B} $$

where $\theta$ is the gravimetric soil water content (%), S is the soil suction (bar), and A is the SWHC.

Employing the equation above, we measured soil water content at a potential of − 20 bar for the wilting point, − 0.3 bar for the field capacity, and − 10 bar for the critical point of the rapid available water and slow available water. The rapid available water content was calculated as the soil water content under suction from − 0.3 to − 10 bar, the slow available water content was calculated as the soil water content under suction from − 10 to − 20 bar, the total available water content was calculated as the sum of rapid available water content and rapid available water content, and the unavailable water content was calculated as the soil water content under suction of − 20 bar^16,24^.

### Data analysis

One-way ANOVA was conducted to test the significance of differences in the soil water characteristics among the different vegetation restoration stages. Statistical analyses were performed with the SPSS software package (Version 16.0 for Windows; SPSS, Inc., USA). The regression equations were fit with a modified three parameter exponential decay using SigmaPlot version 10.0, from Systat Software, Inc., San Jose California USA, www.systatsoftware.com. *P* < 0.05 was considered statistically significant.

## Results

### SWA

**Figure 3:**
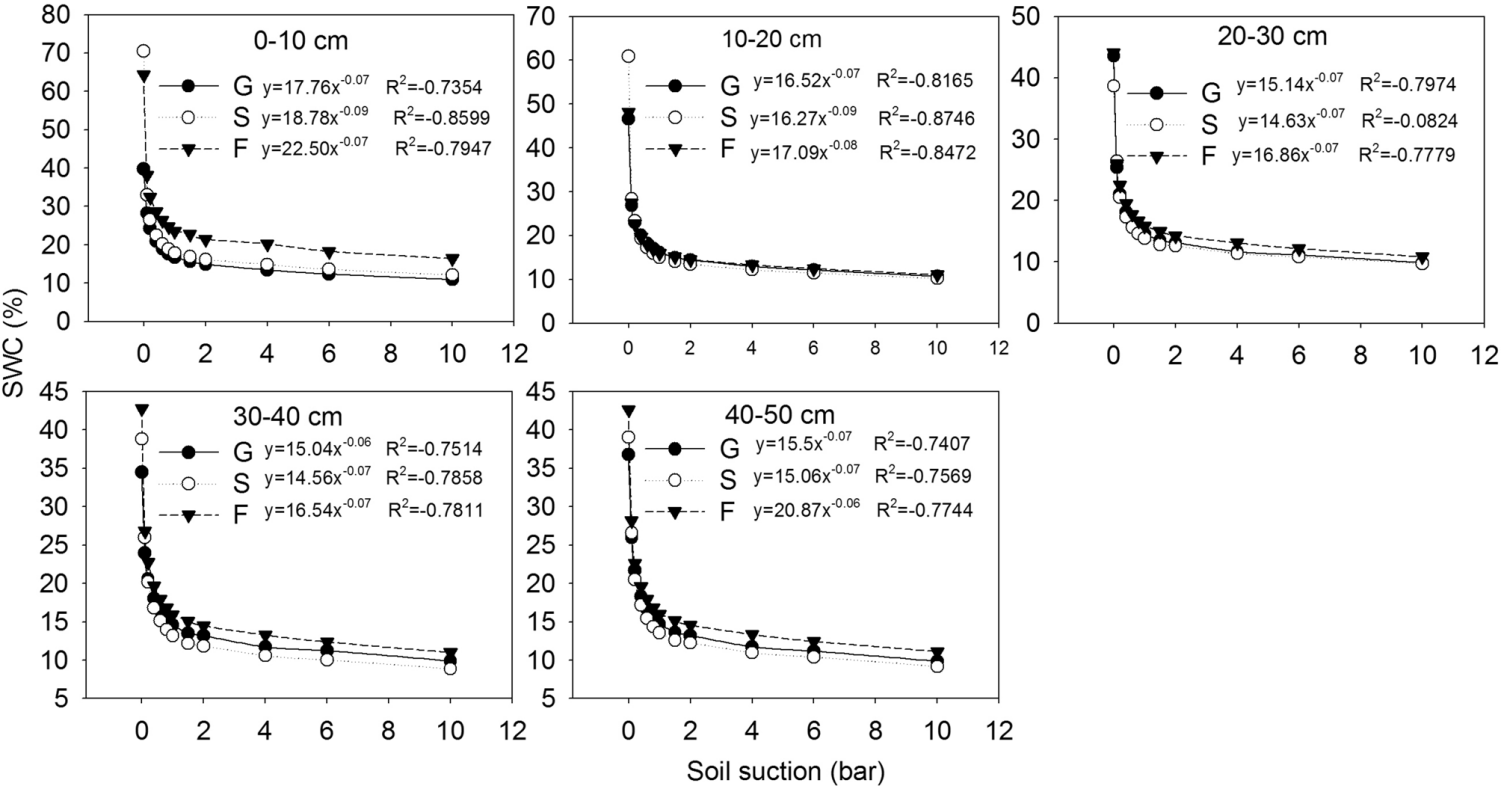
Soil water characteristic curves at different soil depths (0–10 cm, 10–20 cm, 20–30 cm, 30–40 cm and 40–50 cm) at different vegetation restoration stages. G, S and W are defined as in Table 1.

**Figure 4:**
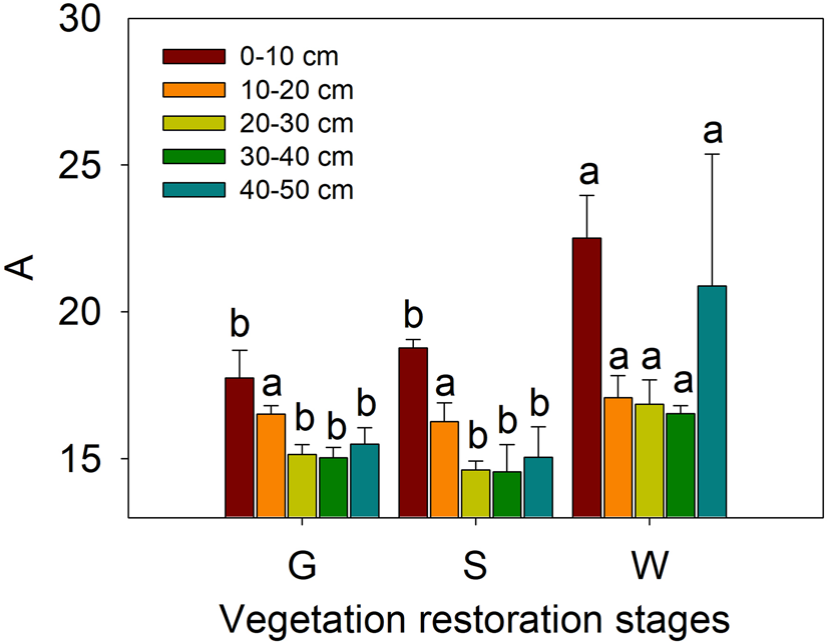
Soil water characteristic parameter A in the different soil layers at different vegetation restoration stages (see Table 1). The values are in the form of means ± SEs, and the sample size (n) is 5. The different lowercase letters above the bars indicate significant differences within a soil layer among the different vegetation restoration stages (*P* < 0.05).

Soil water characteristic curves at different soil depths (0–10 cm, 10–20 cm, 20–30 cm, 30–40 cm and 40–50 cm) in different vegetation restoration stages are shown in Fig. 3. With increasing suction, the soil water content in each soil layer decreased, and when the suction reached 2 bar, the decreasing trend of the soil water content stabilized at all vegetation restoration stages. During the dehydration process, in the soil layers above 20 cm, the soil water characteristic curve was highest at the grassland site, second highest at the shrubland site, and lowest at the woodland site. However, below 20 cm, the curve was lowest at the shrubland site. The values of the soil water characteristic parameter A in the different soil layers at the different vegetation restoration stages are shown in Fig. 4. Parameter A was significantly higher at the woodland site than at the grassland and shrubland sites in all of the soil layers except the 10–20 cm layer (*P* < 0.05), and there was no significant difference in this parameter between the grassland and shrubland in any of the soil layers from 0 to 50 cm (*P* > 0.05), this indicated that SWHC was higher at the woodland site than the grassland and shrubland, and there was no significant difference between the latter two sites.

**Figure 5:**
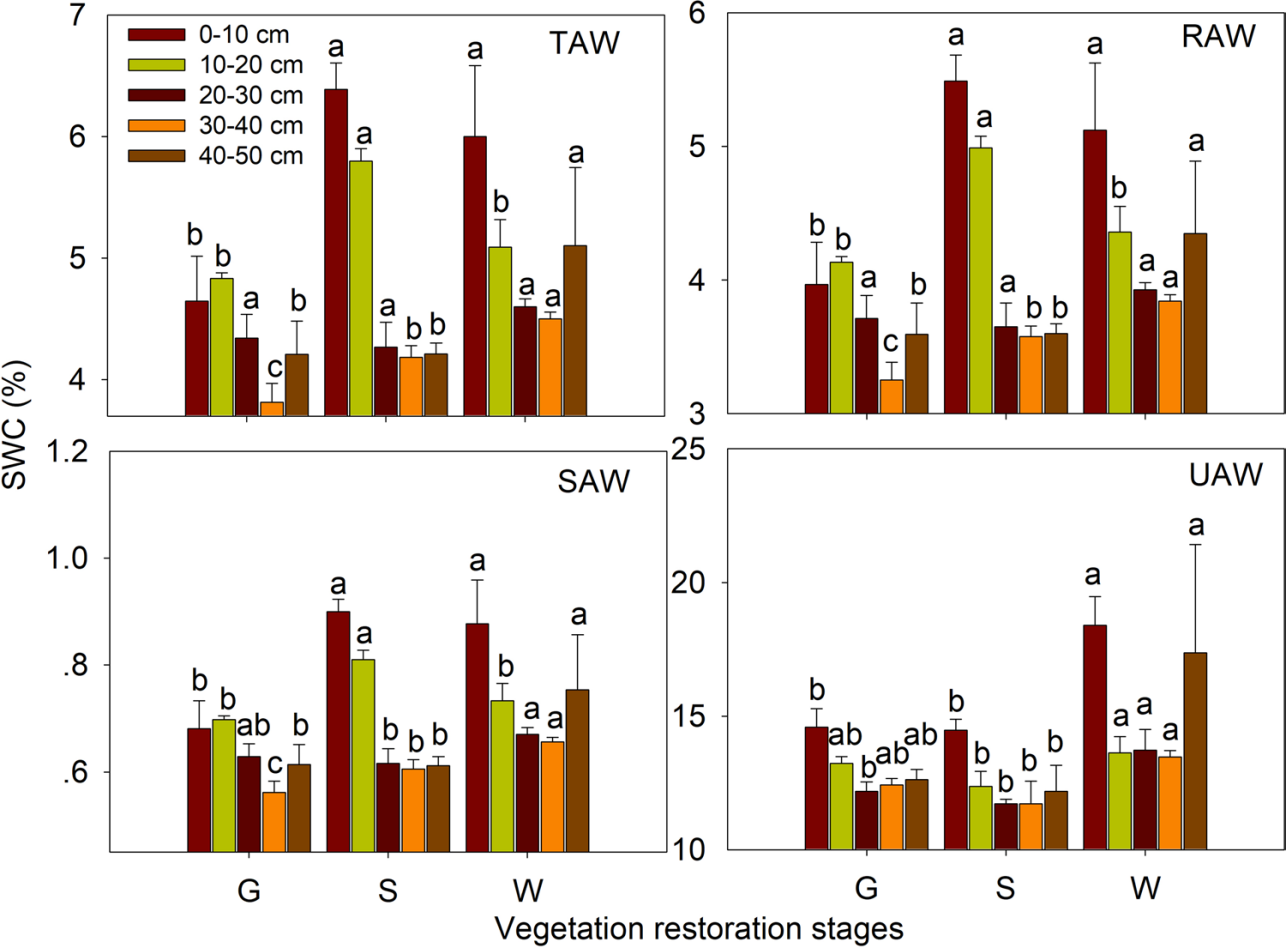
Total available water content (TAW), rapid available water content (RAW), slow available water content (SAW) and unavailable water content (UAW) in the different soil layers at different vegetation restoration stages (see Table 1). The values are in the form of means ± SEs, and the sample size (n) is 5. The different lowercase letters above the bars indicate significant differences within a soil layer among the different vegetation restoration stages (*P* < 0.05).

Total available water content, rapid available water content, slow available water content and unavailable water content in the different soil layers at the different vegetation restoration stages are shown in Fig. 5. In the 0–10 cm soil layer, the total available water content at the grassland site was significantly lower than the corresponding contents at the shrubland and woodland sites (*P* < 0.05), and no significant difference was identified between shrubland and woodland. In the 10–20 cm soil layer, total available water content at the shrubland site was obviously higher than the corresponding contents at the two other sites representing different vegetation restoration stages (*P* < 0.05), and no difference was detected between grassland and woodland. In the 20–30 cm soil layer, no significant difference was found among any of the three vegetation restoration stages; in the 30–40 cm soil layer, total available water content increased clearly from grassland to shrubland to woodland (*P* < 0.05). In the 40–50 cm soil layer, total available water content was markedly higher at the woodland site than at the grassland and shrubland sites (*P* < 0.05), and no significant difference between the latter two sites, representing different vegetation restoration stages, was found, in general, the SWA was higher at the woodland site than the grassland and shrubland, and there was no significant difference between the latter two sites, the trend of SWA was similar to the SWHC. Furthermore, the variations of rapid available water content and slow available water content in the 0–50 cm soil layer were generally similar to those of total available water content. However, unavailable water content in the 0–50 cm soil layer was highest in woodland among the three land use types (*P* < 0.05), and there was no clear difference between the grassland and shrubland.

### Soil properties related to SWA

**Figure 6:**
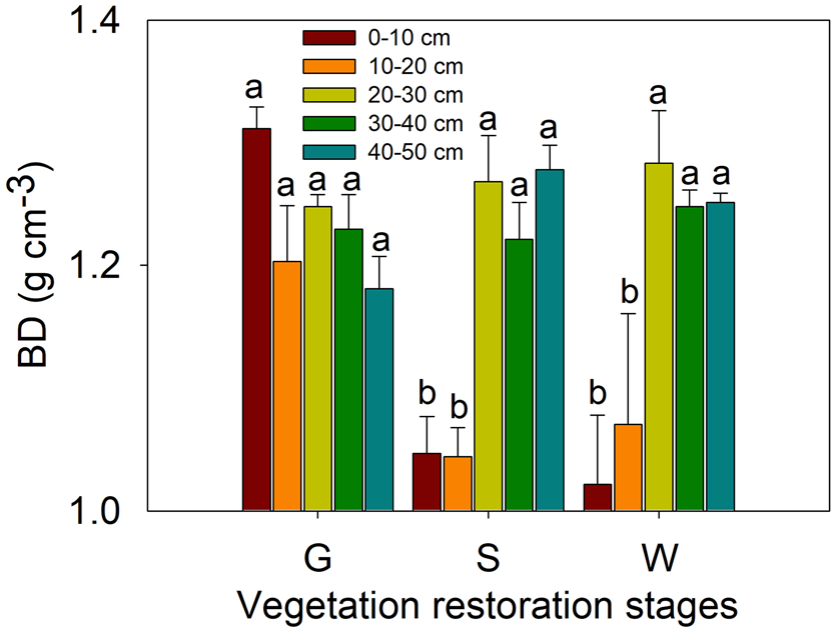
Soil bulk densities (BDs) in the different soil layers at different vegetation restoration stages (see Table 1). The values are in the form of means ± SEs, and the sample size (n) is 5. The different lowercase letters above the bars indicate significant differences within a soil layer among the different vegetation restoration stages (*P* < 0.05).

**Figure 7:**
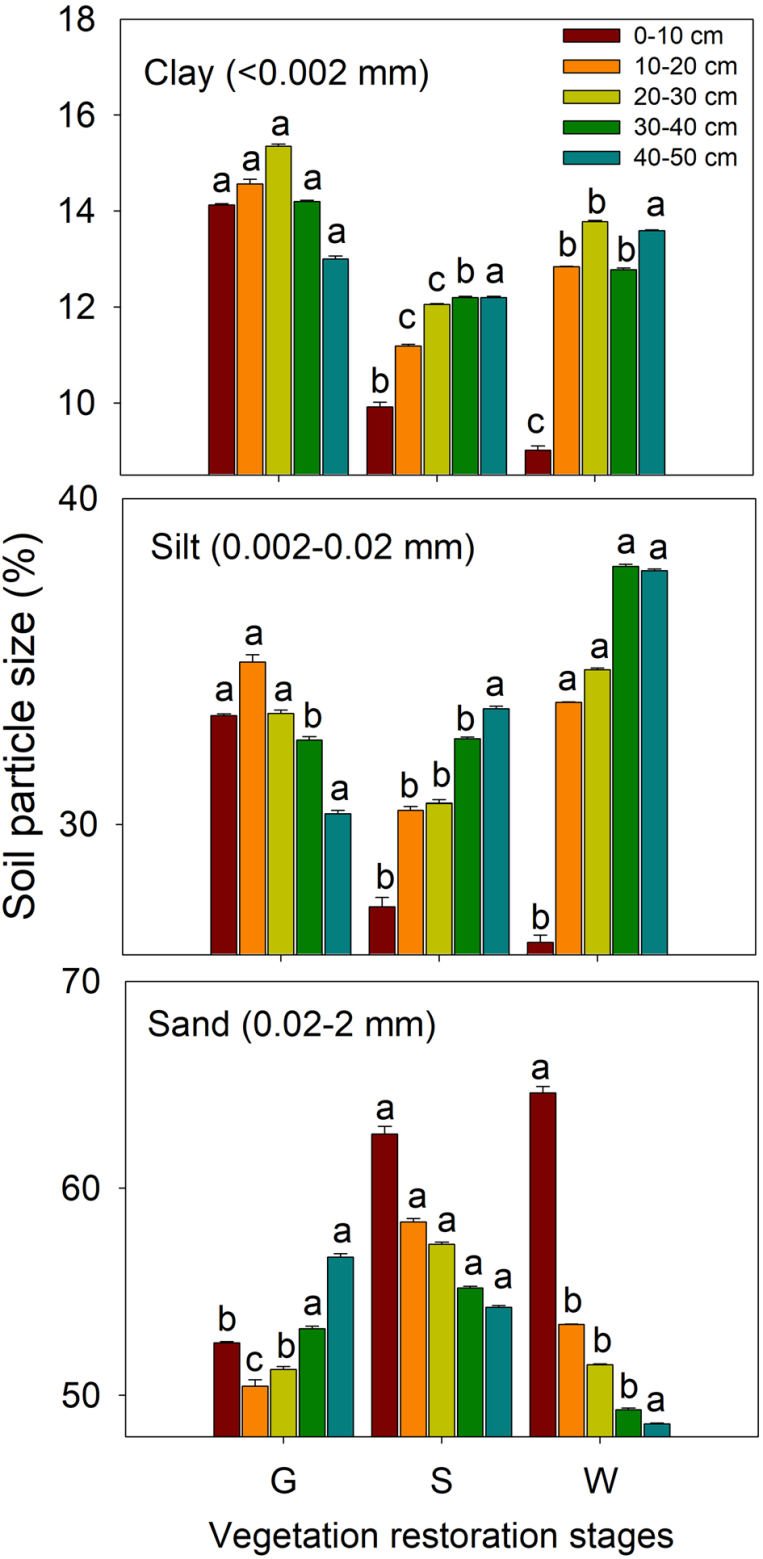
The contents of clay (< 0.002 mm), silt (0.002–0.02 mm) and sand (0.02–2 mm) in the different soil layers at different vegetation restoration stages (see Table 1). The values are in the form of means ± SEs, and the sample size (n) is 5. The different lowercase letters above the bars indicate significant differences within a soil layer among the different vegetation restoration stages (*P* < 0.05).

The values of soil BD in the different soil layers at the different vegetation restoration stages are shown in Fig. 6. In the 0–10 and 10–20 cm soil layers, BD was significantly higher at the grassland site than at the shrubland and woodland sites (*P* < 0.05), and no obvious difference among the three vegetation restoration stages was observed in the soil layers below 20 cm. The contents of clay (< 0.002 mm), silt (0.002–0.02 mm) and sand (0.02–2 mm) in the different soil layers at the different vegetation restoration stages are shown in Fig. 7. The distribution of soil particle size significantly varied among the different vegetation restoration stages. In the 0–10 cm soil layer, the clay and silt contents decreased significantly from grassland to shrubland to woodland (*P* < 0.05); however, sand showed the opposite pattern, and both silt and sand varied slightly from shrubland to woodland (*P* > 0.05). In the 10–20 and 20–30 cm soil layers, clay content was highest at the grassland site, followed by the woodland site, and lowest at the shrubland site, and silt content was lowest and sand content was highest in shrubland. In the 30–40 cm soil layer, clay content was similar between the shrubland and woodland sites and lower at both these sites than at the grassland site (*P* < 0.05). In addition, in this layer, both the silt content and sand content were similar between the grassland and shrubland; the former was highest and the latter was lowest in the woodland. In the 40–50 cm soil layer, no clear difference was evident among the different vegetation restoration stages.

**Figure 8:**
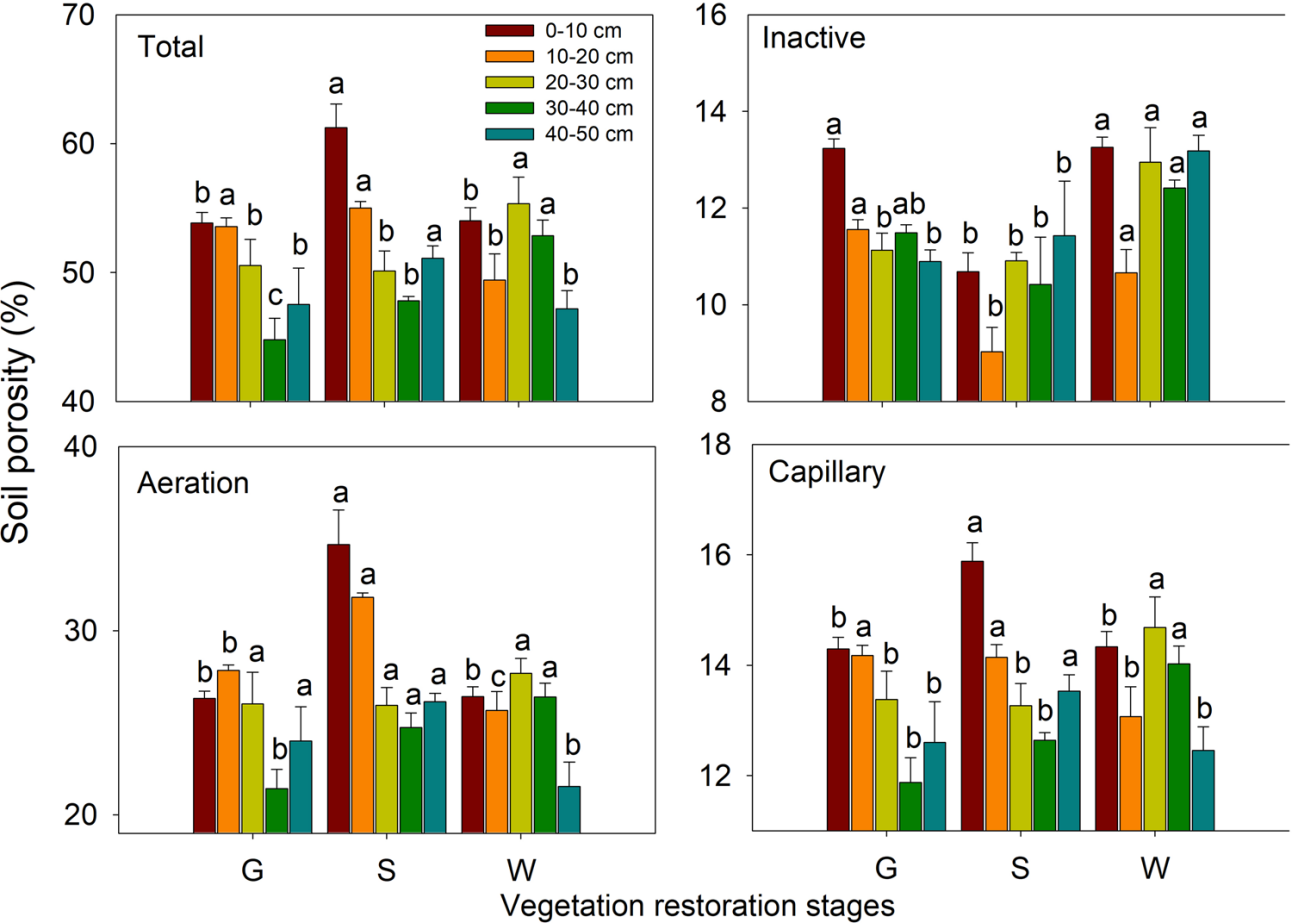
Soil total porosity, inactive porosity, aeration porosity and capillary porosity in the different soil layers at different vegetation restoration stages (see Table 1). The values are in the form of means ± SEs, and the sample size (n) is 5. The different lowercase letters above the bars indicate significant differences within a soil layer among the different vegetation restoration stages (*P* < 0.05).

The soil porosity parameters in the different soil layers at the different vegetation restoration stages are shown in Fig. 8. In the 0–10 cm soil layer, soil total porosity, aeration porosity and capillary porosity were higher in shrubland than in grassland and woodland (*P* < 0.05), whereas inactive porosity exhibited the opposite pattern, being lowest in shrubland. None of these parameters significant differed between grassland and woodland. In the 10–20 cm soil layer, total porosity and capillary porosity were lower at the woodland site than at the grassland and shrubland sites. Furthermore, in this layer, inactive porosity was lowest at the shrubland site, and aeration porosity was highest at the shrubland site, followed by the grassland site, and lowest at the woodland site. In the 20–30 cm soil layer, total porosity, inactive porosity and capillary porosity were higher in woodland than in grassland and shrubland, while aeration porosity varied slightly among the different vegetation restoration stages (*P* > 0.05). In the 30–40 cm soil layer, total porosity, inactive porosity, aeration porosity and capillary porosity were highest in woodland and lowest, except for inactive porosity, in grassland. In the 40–50 cm soil layer, total porosity and capillary porosity were higher in shrubland (*P* < 0.05) than in the other land use types, and no significant difference was observed between grassland and woodland. In this layer, inactive porosity was highest and aeration porosity was lowest in woodland; however, neither obviously differed between grassland and shrubland. In general, the soil porosity was higher in the shrubland and woodland than that in the grassland.

### Relationship between SWA and soil properties

Pearson’s correlation coefficients among the soil water characteristic parameter A and soil properties are shown in Table 2. Across the three vegetation restoration stages, in the 0–50 cm soil layer, parameter A, total available water content, rapid available water content, slow available water content, and unavailable water content showed highly significant and positive relationships (*P* < 0.01) (Table 2). Total available water content, rapid available water content, slow available water content and unavailable water content were significantly and positively related to each other (*P* < 0.05). Furthermore, the relationship between A and inactive porosity was significant and positive (*P* < 0.05). Both total available water content and rapid available water content were strongly and positively related to both aeration porosity and capillary porosity (*P* < 0.01) and highly significantly and negatively related to clay content and BD (*P* < 0.01). Rapid available water content was positively related to sand content (*P* < 0.05), total porosity (*P* < 0.01), aeration porosity (*P* < 0.05) and capillary porosity (*P* < 0.01) and negatively related to clay content and BD (*P* < 0.01). Unavailable water content was highly and positively related to inactive porosity (*P* < 0.01). Highly significant and positive relationships were observed among clay content, silt content and BD (*P* < 0.01); however, sand content and BD were highly significantly negatively related (*P* < 0.01) (Table 2).

**Table 2:** Pearson’s correlations between soil water characteristic parameters and properties.

	A	TAW	RAW	SAW	UAW	Clay	Silt	Sand	TP	IP	AP	CP
A	1											
TAW	0.724**	1										
RAW	0.713**	1.000**	1									
SAW	0.787**	0.995**	0.994**	1								
UAW	0.986**	0.597*	0.585*	0.672**	1							
Clay	− 0.453	− 0.686**	− 0.687**	− 0.678**	− 0.358	1						
Silt	− 0.191	− 0.484	− 0.487	− 0.462	− 0.103	0.712**	1					
Sand	0.302	0.595*	0.597*	0.576*	0.206	− 0.871**	− 0.965**	1				
TP	0.340	0.721**	0.724**	0.695**	0.219	− 0.453	− 0.347	0.412	1			
IP	0.576*	− 0.053	− 0.066	0.035	0.682**	0.166	0.320	− 0.286	0.011	1		
AP	0.099	0.696**	0.705**	0.638*	− 0.055	− 0.488	− 0.446	0.494	0.920**	− 0.381	1	
CP	0.353	0.679**	0.682**	0.660**	0.244	− 0.422	− 0.318	0.380	0.994**	0.095	0.879**	1
BD	− 0.441	− 0.796**	− 0.799**	− 0.775**	− 0.317	0.711**	0.651**	− 0.720**	− 0.376	0.402	− 0.510	− 0.322

*A* soil water characteristic parameter, *TAW* total available water content, *RAW* rapid available water content, *SAW* slow available water content, *UAW* unavailable water content, *TP* total porosity, *IP* inactive porosity, *AP* aeration porosity, *CP* capillary porosity, *BD* bulk density.

*Correlation is significant at the 0.05 level (2-tailed).

**Correlation is significant at the 0.01 level (2-tailed).

## Discussion

In our study, soil water characteristic curves were generated by the dehydration process, and the curve for the grassland site was higher than the curves for the shrubland and woodland sites. This finding indicated that soil water content in 0–50 cm soil layers at the grassland site was higher than that at the shrubland and woodland sites; Yang et al.^25^ obtained similar findings. This difference may be because soils at the grassland site had higher clay and silt contents and a lower sand content than the soils at the shrubland and woodland sites (Fig. 7); clay particles absorb water more than sand particles^7^. Another reason may be that the soils at the woodland site tended to have lower water contents because of the higher root densities of trees and the resulting greater transpiration ability compared with that of grasses^20^.

The type of land use pattern affects the balance of soil material composition and SWHC. SWHC is affected by soil texture and organic carbon content and soil characteristics, such as pore characteristics. In this study, the soil water characteristic parameter A (Fig. 4) was highest at the woodland site, indicating that SWHC in the 0–50 cm soil layer was strongest at this site. This finding might have been due to a greater amount of soil water absorbed by roots in woodland, as there are higher root densities in woodland than in grassland^20^. Furthermore, there may have been more soil water stored within inactive pores in grassland, with inactive porosity increasing and BD decreasing with increasing A (Table 2). A was lower, i.e., the SWHC was weaker, in the grassland sites. Another reason is that the scarce vegetation at the grassland site may have led to the compaction of the surface soil layer, causing precipitation to be readily converted to surface runoff and decreasing the permeation of precipitation into the soil^26^. Thus, inactive porosity and BD may be the key soil properties affecting SWHC at different vegetation restoration stages.

The SWA status can limit the root and microbial activities^27,28^ and affect substrate availability, belowground carbon allocation and plant growth; accordingly, it exerts direct or indirect effects on microbial respiration and plant roots^17^. From the grassland to the woodland, the available water content was drastically increased in 0–50 cm soil layers, the reason is that the clay content was decreasing and the sand content was increasing during the vegetation restoration, this finding was agreement with the results of Li et al.^15^. Besides, the available water content correlated positively with sand content and negatively with clay content, this indicated that soil texture was improved during the vegetation restoration^29^, which caused the increase in SWA. This is also because of the increasing soil porosity in the long-term vegetation restoration. The increase of SWA was induced by the increase of the soil porosity which can improve the soil water storage and retaining capacity^30^, these findings are in agreement with those of Udawatta and Anderson^31^. Soil water storage can be drastically affected by the change of vegetation types, for instance, soil water content significantly decreased because of the increase of tree biomass^32^. Along with the vegetation restoration, the soil water storage remarkably decreased^33^ due to the decrease of soil water content which can be significantly affected by the vegetation types and structures^34^. Different vegetation types have the different root systems and transpiration which can lead the large spatial variation. Besides, soil moisture reduced remarkably after vegetation restoration, and it was varied significantly in the deep soil layers and slightly in the surface soil layers among different vegetation types^35^. The study showed that the soil water storage reduced drastically during the vegetation restoration above 500 cm soil layers due to the decreasing soil water content. The soil water storage exhibits a significant positive relationship with the soil moisture. In our previous study, BD in the 0–50 cm soil layers declined during vegetation restoration, from this we can find the soil physical properties were polished up partly in this process^36^. Nevertheless, BDs in the surface soil layers had no evident differences between the different vegetation types (the succession ages were < 30 years), showing that soil physical properties would never be improved until the natural vegetation succession ages were more than 30 years^29^. Furthermore, the change in soil sand content was consistent with that of BD; however, the change in soil porosity was the opposite. This finding indicates that the soil degradation caused by the desertification is not easy to turn back rapidly^36^. In addition, the decrease in BD from grassland to shrubland to woodland (Fig. 6) improved the physical properties of the soil in the 0–50 cm layer to some extent, which is consistent with the findings of Wang et al.^36^; these improvement in turn increased the SWA, and a highly significant negative relationship was observed between BD and each of total available water content, rapid available water content and slow available water content (Table 2). Therefore, soil texture, porosity and BD are the crucial factors affecting SWA.

As the land serves as the carbon source and the carbon sink, the soil function was altered by the change in land use type over the course of the natural vegetation restoration^37^. In previous research, the soil organic carbon (SOC) increased gradually following vegetation succession from grassland to shrubland to woodland, and soil water storage showed a negative relationship with soil organic storage^38^. Zhao et al.^30^ reported that the importance of organic matter is significant during the process of soil aggregate formation, as it acts as the coagulating substance, and different soil solid states with different porosities result from different soil aggregate bindings caused by different organic matter components. With the accumulation of soil organic matter over the course of natural vegetation restoration, the soil porosity characteristics are inevitably affected, as reported by Deng et al.^37^. Furthermore, Zhao et al.^30^ demonstrated that soil porosity is one of the most important factors affecting the soil reservoir following the natural vegetation restoration on the Chinese Loess Plateau. Soil N and P also showed negative relationships with soil water storage; in previous research, such patterns were attributed to increases in leaf N and P, which are transformed to soil N and P, over the course of vegetation restoration^33^. Luo et al.^39^ showed that N dynamics are the main factor regulating soil carbon sequestration; furthermore, one of the most common factors limiting crop production is N. Therefore, soil C, N and P are important chemical factors affecting the soil water characteristics during the process of vegetation restoration, with soil physical properties also affecting these characteristics.

## Conclusions

On the Loess Plateau, the soil water characteristics varied from grassland to shrubland to woodland, representing different vegetation restoration stages. The SWHC, as measured by the soil water characteristic parameter A derived from the soil water characteristic curves, was higher at the woodland site than the grassland and shrubland, and there was no significant difference between the latter two sites, the trend of SWA was similar to the SWHC. From grassland to woodland, the soil physical properties in the 0–50 cm soil layer partially improved, BD was significantly higher at the grassland site than at the shrubland and woodland sites, the clay and silt contents decreased significantly from grassland to shrubland to woodland and sand content showed the opposite pattern, the soil porosity was higher in the shrubland and woodland than that in the grassland. Soil texture, porosity and BD were the key factors affecting SWHC and soil water availability. The results of this study provide insight into the effects of vegetation restoration stage on local hydrological resources and can inform soil water management and land use planning on the Chinese Loess Plateau.
